# The 6 degrees of curriculum integration in medical education in the United States

**DOI:** 10.3352/jeehp.2024.21.15

**Published:** 2024-06-13

**Authors:** Julie Youm, Jennifer Christner, Kevin Hittle, Paul Ko, Cinda Stone, Angela D. Blood, Samara Ginzburg

**Affiliations:** 1 University of California, Irvine School of Medicine, Medical Education, Irvine, CA, USA; 2 Department of Pediatrics, School of Medicine and School of Health Professions, Baylor College of Medicine, Houston, TX, USA; 3 School of Pharmacy and Pharmaceutical Sciences, The State University of New York at Buffalo, Buffalo, NY, USA; 4 Department of Emergency Medicine, Indiana University School of Medicine, Indianapolis, IN, USA; 5 Pre-Clerkship and Curricular Management, University of Arizona College of Medicine-Phoenix, Phoenix, AZ, USA; 6 Association of American Medical Colleges, Washington, DC, USA; 7 Donald and Barbara Zucker School of Medicine at Hofstra/Northwell, Hempstead, NY, USA; Hallym University, Korea

**Keywords:** Accreditation, Curriculum, Medical education, Patient care

## Abstract

Despite explicit expectations and accreditation requirements for integrated curriculum, there needs to be more clarity around an accepted common definition, best practices for implementation, and criteria for successful curriculum integration. To address the lack of consensus surrounding integration, we reviewed the literature and herein propose a definition for curriculum integration for the medical education audience. We further believe that medical education is ready to move beyond “horizontal” (1-dimensional) and “vertical” (2-dimensional) integration and propose a model of “6 degrees of curriculum integration” to expand the 2-dimensional concept for future designs of medical education programs and best prepare learners to meet the needs of patients. These 6 degrees include: interdisciplinary, timing and sequencing, instruction and assessment, incorporation of basic and clinical sciences, knowledge and skills-based competency progression, and graduated responsibilities in patient care. We encourage medical educators to look beyond 2-dimensional integration to this holistic and interconnected representation of curriculum integration.

## Graphical abstract

[Fig f2-jeehp-21-15]

## Introduction

### Background/rationale

There are both implicit and explicit expectations that medical schools integrate content within their curriculum. The traditional “2+2” curricular structure, with 2 years of basic science followed by 2 years of clinical science, has been promoted since the publication of the Flexner Report in 1910 [1]. The recommendation to integrate within and across these 2-year phases has been popular for the past 5 decades as the focus on providing trainees skills directly related to patient care took precedence over systematic, yet separate, knowledge of the basic and clinical sciences [2]. When looking at a few key measures of curricular integration, one might assume that many medical schools are heeding this recommendation. In the annual Medical School Graduation Questionnaire administered by the Association of American Medical Colleges in 2023, 82% of graduating medical students agreed that their basic science coursework had sufficient illustrations of clinical relevance, and 85% agreed that their required clinical experiences integrated basic science content [3]. Nonetheless, it is unclear if these are the appropriate measures by which effective curriculum integration should be evaluated.

To support curriculum evaluation, the Liaison Committee on Medical Education (LCME) requires MD (Doctor of Medicine)-granting medical education programs in the United States to describe the process used to determine the horizontal and vertical integration of curriculum content in an accreditation element focused on curricular design, review, and revision/content monitoring (Element 8.3) [4]. An analysis of variables leading to severe action decisions by the LCME found that noncompliance with this element has a significant correlation to a severe action decision [5]. This leads to an undeniable motivation for medical schools to achieve horizontal and vertical integration in their medical education programs and understand how to implement this concept.

### What is curriculum integration?

Despite the explicit expectations and accreditation requirements for an integrated curriculum, there appears to be less clarity around an accepted definition, guidelines, and best practices for medical educators to implement an integrated curriculum. “I know it when I see it” is how Justice Potter Stewart of the United States Supreme Court famously acceded to define obscene materials in the case of Jacobellis v. Ohio (1964) [6]. This phrase may be relatable to medical educators who feel comfortable with the concept of curriculum integration, but fail to find the exact words that define or describe what they know intuitively.

We consulted the Glossary of Terms for LCME Accreditation Standards and Elements [7] and found that it did not include a definition for curriculum integration, although the concept is mentioned in the glossary entry for “coherent and coordinated medical curriculum”:

“Coherent and coordinated medical curriculum: The design of a complete medical education program, including its content and modes of presentation, to achieve its overall educational objectives. Coherence and coordination include the following characteristics: (1) the logical sequencing of curricular segments, (2) coordinated and integrated content within and across academic periods of study (i.e., horizontal and vertical integration), and (3) methods of instruction and student assessment appropriate to the student’s level of learning and to the achievement of the program’s educational objectives (Element 8.1)” [4].

We also conducted a literature review using the following search strategy: definitions of vertical and horizontal integration in education (general K-12, higher education) or medical education literature; existing visual representations of vertical and horizontal integration in curriculum in education (general K-12, higher education) or medical education literature; representation of longitudinal integrated clerkships in medical education curriculum; and representation of content threads and curricular sequencing in curriculum found via Google image searches using the site limiter “.edu”. Studies that focused primarily on the impact of curriculum models and studies that focused primarily on faculty or student satisfaction with curriculum models were excluded.

The literature review yielded multiple definitions for curriculum integration. For example, Malik and Malik [8] explicitly suggest the following definitions for horizontal and vertical integration:

“The horizontal integration is integration between parallel disciplines, such as anatomy, physiology and biochemistry or medicine, surgery and therapeutics traditionally taught in the same phase of the curriculum.”

“The vertical integration is integration between disciplines traditionally taught in different phases of the curriculum. It can occur throughout the curriculum with the basic medical and clinical sciences beginning together in the early years of the curriculum and continuing until the later years.”

These definitions focus on *disciplines*, such as anatomy physiology, medicine, surgery, as well as *timing* (i.e., between different phases). While the proposed definitions align with interpretations of horizontal and vertical integration within medical education [1,9-12] and more broadly in higher education [13,14], further exploration of the literature highlights that the definitions around integration remain loosely defined [9]. Additional attributes that have been used to define integration beyond *content/discipline* and *timing* include the *deliberate combination of basic sciences and clinical sciences* [15], *a knowledge and skills-based progression* from “novice” to “competence” [10], and *graduated responsibilities in patient care* [1]. Determining which of these attributes to consistently apply when planning for integration for the greatest educational outcomes is open for interpretation and the aim of our work here.

### Objectives

To address the lack of consensus around the definition of integration including and expanding upon vertical and horizontal integration, we identified the objectives of this article as follows: (1) construct a definition for curriculum integration based on the literature; (2) illustrate applications of the definition for medical educators; and (3) generate discussion around curriculum integration as we look to the future using the proposed definition.

## Six degrees of curriculum integration in medical education

One reason that curriculum integration may have been historically challenging to define is that a singular definition is not the most suitable approach to illustrating the concept, given the multiple attributes it may represent, such as the level of competency, variation across time, multiple content areas, and so forth [1,7-14]. Harden’s “integration ladder” [12] recognizes this and provides a framework that defines curriculum stages along the progression from least integrated (“isolation”) to most integrated (“transdisciplinary”) [12]. Ultimately, the case for integration is not just integration for its own sake. Integration is instead a strategy for turning knowledge into action [16] to improve practice and patient outcomes. Thus, a more modern definition of curriculum integration that makes direct connections between integration of content and assessment of abilities would help to achieve this.

**Fig. 1.**
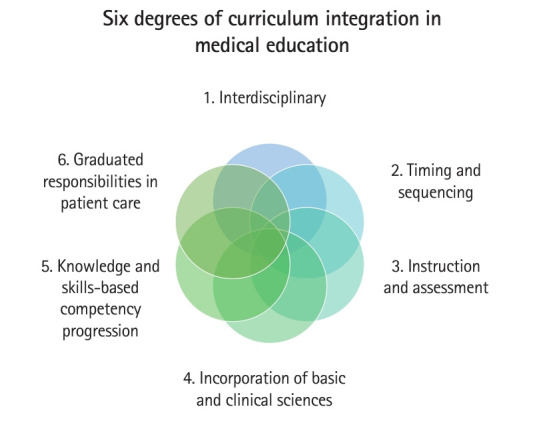
Six degrees of curriculum integration in medical education.

In turn, we propose a “model” instead of a simple “definition” of curriculum integration. The basis of this proposed model is the results from the literature review described above to make tangible the concept of curriculum integration. This new model, presenting 6 degrees of curriculum integration, incorporates the following concepts (Fig. 1): (1) interdisciplinary, (2) timing and sequencing, (3) instruction and assessment, (4) incorporation of basic and clinical sciences, (5) knowledge and skills-based competency progression, and (6) graduated responsibilities in patient care.

As with the 6 degrees of freedom that describe the ways in which a rigid object can move through 3-dimensional space and the concept of 6 degrees of separation that suggests that humans are connected through a chain of 6 people, this model implies a holistic, comprehensive and interconnected representation of curriculum integration, incorporating and expanding upon the 2 dimensions of horizontal and vertical by defining and including other attributes of integration as described in the literature. Further, we add the guiding principle that comes from the LCME, of emphasis on coordination that implies the integration of curriculum as intentional—that is, as a design choice where the 6 degrees are applied deliberately, rather than one that simply happens temporally or coincidentally.

### First degree: interdisciplinary

The first degree of curriculum integration is the combination of 2 or more disciplines into an interdisciplinary curricular segment. A broader look into the definitions by Malik and Malik [8] and other authors in the field reveals that they commonly support a structural organization around the attribute of *content/discipline*. A discipline is a field of study [17]. Medical education curricula are composed of many disciplines that reflect basic and clinical sciences, such as anatomy, physiology, surgery, and neurology. Harden [12] identifies an interdisciplinary approach to integration whereby “the subjects and disciplines give up a large measure of their own autonomy” (p. 555). Integration of content in this way supports the idea of “cognitive conceptual coherence,” the creating of a mental model that helps learners organize a collection of information in their minds [18]. This in turn allows students to find relevance of the content to promote their retention of information and improve their ability to transfer knowledge to the situations they encounter [19].

### Second degree: timing and sequencing

The second degree of curriculum integration is the presentation of content across time and within curricular sequences. Definitions by Malik and Malik [8] and other authors in the field reveal an explicit attribution to the *timing and sequencing* of integration—that is, within or across an academic period of study. Vergel et al. [20] similarly discuss “macro levels” of integration where learners interact with content “at the same level” and “as the program progresses” (p. 247). The definitions by Malik and Malik [8] (presented above) bring together the attribute of content/discipline with that of timing and sequencing to provide a familiar 2-dimensional representation of “parallel disciplines within the same phase” and “disciplines taught in different phases”. However, it is important to keep in mind that timing and sequencing also bring a structural perspective to curriculum integration that extends beyond curricular phases to include program, course, and session-level considerations as well [16,18,19].

### Third degree: instruction and assessment

The third degree of curriculum integration is the implementation of appropriate instructional and assessment methods that support learning of the integrated content. The integration of curriculum benefits from instructional and assessment methods that guide learners to build upon mental models from prior courses/knowledge and engage in critical thinking to solve clinically relevant problems. *Instructional methods* such as problem-based learning, team-based learning, and simulation promote critical thinking through rich real-world contexts that inherently include the interconnectedness of curricular content from multiple disciplines and phases.

Accordingly, *assessment methods* that complement a constructivist instructional approach are necessary to determine student achievement of competencies in integrated content areas. Assessment methods that support such learning focus on formative assessments over summative methods and opportunities for ongoing feedback, self-assessment, and reflection may be most relevant [16]. A “hidden curriculum” that emphasizes the acquisition of facts rather than the application of concepts has presented a barrier to address assessment of integration [19]. This emphasis on knowledge and facts leads to the common use of multiple-choice questions. While multiple-choice questions do provide value in assessment, they do not capture a demonstration of knowledge in action [16] and the nuances of learning from an integrated curriculum. Alternative assessment methods that could be considered include essay-based exams, concept maps, and portfolios [16].

### Fourth degree: incorporation of basic science and clinical science

The fourth degree of curriculum integration is the *deliberate combination of the basic sciences and the clinical sciences* [15]. The traditional 2+2 structure allocated time in a medical education program to reflect these foundational fields in the study of medicine, but fell short in promoting the blending of these sciences rather than a sequential presentation.

Though the degrees of interdisciplinary and timing/sequencing often imply the integration of the basic and clinical sciences, we include this here as a formal concept in the model to explicitly bring attention to blending content in this way. More importantly, we recognize that when a physician is seeing a patient in clinic, and formulating their clinical decision process, they are not thinking in terms of basic science or clinical science separately. The physician is actively integrating all elements of their knowledge base and competencies in a holistic manner to make evidence-based decisions about patient care. Thus, educating our learners to practice medicine with a holistic perspective will better prepare them as future clinicians.

### Fifth degree: knowledge and skills-based progression

The fifth degree of curriculum integration is the focus on a *knowledge and skills-based progression*. “See one, do one, teach one” is a familiar adage in medical education that describes a traditional, stepwise approach to teaching clinical skills [21]. Though the applicability of this teaching approach is evolving, the concept of a knowledge and skills-based progression from “novice” to “competence” has appeared in the literature for some time [10] and offers another degree of approaching curriculum integration. Integration of curricular content benefits from the coordination of opportunities for students to learn in a progressive fashion so that they are gaining new knowledge instead of a repetition of prior knowledge and using that knowledge to advance their clinical skills over time.

Knowledge and skills-based progression can support a competency-based medical education approach that tracks learner outcomes organized around a framework of competencies for which students should achieve mastery [22]. Achieving the expected competencies by the time of graduation ensures that medical students are prepared to meet the expectations of patients and the profession as they transition to residency. The milestones in residency by specialty and sub-specialty incorporated by the Accreditation Council for Graduate Medical Education [23] provide further attention to the developmental process of producing competent physicians. Therefore, we propose that a combination of knowledge and skills aligning with the developmental process of the learner should be a key consideration for curriculum integration.

### Sixth degree: graduated responsibilities in patient care

The sixth degree of curriculum integration is to ensure learners achieve graduated responsibilities in patient care. The ultimate goal of improving the medical education curriculum through impactful integration is to produce competent physicians [1]. To achieve this, students must have a holistic, systems-based view of the environment in which they will practice as they work towards competence in new areas of medical knowledge, clinical skills, and lifelong learning. We propose that *graduated responsibilities in patient care* is another important degree of curriculum integration and differentiated from the fifth degree of integration, knowledge and skills-based progression, to specifically include the health systems science perspective.

The growth of health systems science (HSS), which is considered to be a third science of medical education [25], offers an example of how systems-based knowledge is necessary to achieve true clinical competence. HSS is “the study and understanding of how care is delivered, how health professionals work together to deliver that care, and how the health system can improve patient care and health care delivery” [24]. HSS has been framed as a synthesis of the basic and clinical sciences with systems-based issues such as quality improvement, health care delivery, and structural and social determinants of health [24]. The HSS lens requires students to engage in a systems-thinking approach and become “system citizens” to understand the interdependency of all the components of healthcare delivery and contribute to the continuous evolution of the healthcare system itself [25] as they progress in their undergraduate medical education.

## Application of the 6 degrees of curriculum integration

Refer to the Supplement 1.

## Conclusion

Current disparate definitions of curricular integration appear to emphasize structural attributes including content/discipline and timing, thereby yielding a 2-dimensional focus on horizontal and vertical integration. While the implementation of horizontal and vertical integration using individual structural attributes has resulted in curricula that better demonstrate for learners the application of basic science to clinical medicine, we suggest that incorporating multiple degrees of integration can support a more holistic approach to educating learners about modern-day complex patient-centered care.

To identify a standardized, comprehensive definition for integration that can be utilized by the community, we herein introduce the model of the 6 degrees of curriculum integration as a conceptual foundation for integration. This model more meaningfully describes how integration can be actualized in medical education programs, thus enhancing educators’ ability to describe, share, and build upon each other’s curriculum practices to which we are accustomed. In this model, each of the 6 degrees is essential in its own right, and it is the intentional synthesis of those parts, coming together to form a whole that is greater than the sum of its pieces, that is its defining feature. We live in an era of multifactorial illnesses, the complex interplay of social determinants of health, and unwieldy healthcare systems. Encouraging medical educators to look beyond 2-dimensional integration will drive the design of educational programs through a macro-perspective that will best prepare physicians of the future to wholly address the current needs of our patients and populations.

Finally, while this work focused on curriculum integration in medical education, we recognize that there are broader implications to curriculum integration in other health professions education that warrant future attention. A limitation of this current work is the narrow scope of its presentation to medical educators. We further acknowledge the need for an accompanying framework for evaluation of how the 6 degrees model can be measured for effectiveness and will continue to explore relevant approaches.

## Figures and Tables

**Figure f2-jeehp-21-15:**
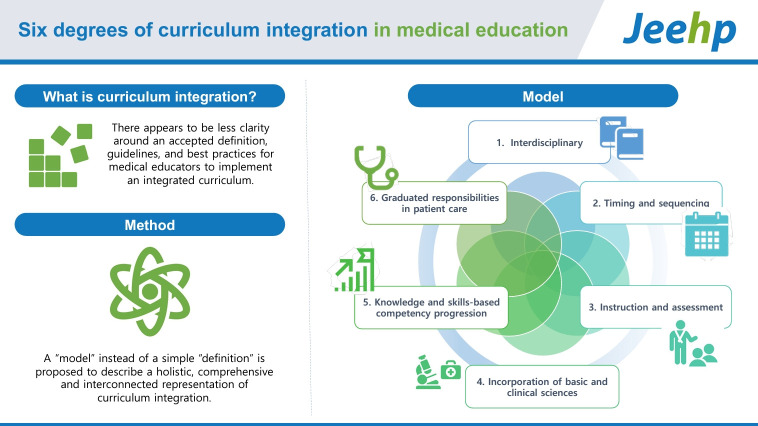


## Supplementary materials

Supplementary files are available from Harvard Dataverse: https://doi.org/10.7910/DVN/4UZDYT

Supplement 1. Application of the 6 degrees of curriculum integration.

Supplement 2. Audio recording of the abstract.
