# Supplementary material for: The 6 degrees of curriculum integration in medical education in the United States
Source: J Educ Eval Health Prof. 2024 Jun 13;21:15. doi: 10.3352/jeehp.2024.21.15 (PMC11261157; doi:10.3352/jeehp.2024.21.15)
Supplement: Supplementary file 1 — Supplement 1. Application of the 6 degrees of curriculum integration. [file jeehp-21-15-suppl1.docx]

**Supplement: Application of the Six Degrees of Curriculum Integration**

In describing the six degrees of curriculum integration model, there is an underlying assumption that a need for integration of a specific curricular content area exists. We share an example of a fictional medical school, Integrations School of Medicine (ISOM), to provide an application to illustrate the model. ISOM has a four-year, systems-based, block curriculum with two years in the pre-clerkship phase and two years in the clinical phase. ISOM is in the state of Arizona and has the goal of integrating learning objectives from the core components of the Arizona Curriculum on Pain and Addiction [1] into its educational program using all six degrees of integration. The focus of their curriculum integration efforts is on the implementation of two learning objectives of the Arizona Curriculum on Pain and Addiction:

1. Describe the interrelated nature of pain and opioid use disorder, including their neurobiology and the need for coordinated management.
2. Employ an integrated, team-based approach to pain and/or addiction care.

To address the first degree of integration, *interdisciplinary*, ISOM included content about the physiologic basis for addiction into sessions taking place in block courses in the disciplines of anatomy, physiology, and neurobiology. This represented the “interdisciplinary” degree of integration, with further interdisciplinary integration occurring through a focus on the behavioral and physiologic manifestations of addiction when taking a history and performing a physical examination during a clinical foundations course in the pre-clerkship phase.

ISOM applied the second degree of integration, *timing and sequencing*, through horizontal integration during the pre-clerkship phase. This was achieved by coordinating content in the pharmacology course on the mechanism of action and side effects of medications used to manage acute and chronic pain with the presentation of social issues in the behavioral science course, including the disparities in pain management in patients with sickle cell disease. Vertical integration was achieved when the public health problems related to opioid misuse and narcan training were included as required content in the surgery, psychiatry, and internal medicine clerkships.

To ensure the third degree of integration, *instruction and assessment*, ISOM looked to pedagogies such as case/problem-based learning (CBL/PBL) and short answer patient-based assessments. CBL/PBL relies upon a case, which by its patient-centered nature includes content from various disciplines, as well as basic, social, and clinical science issues. The pain and addiction curricula during the pharmacology and behavioral science courses at ISOM included a CBL/PBL case of a patient who had low back pain, received opiates for pain relief, and developed opiate dependence. Similarly, a short answer assessment in which a variation of this patient case is presented and students must explain their thinking in writing for how to address the pharmacologic and behavioral health aspects of the case. This case and assessment was also adapted for use and vertically integrated into the surgery, psychiatry and/or internal medicine clerkships

During the design of the pain and addiction content, ISOM engaged both basic and clinical sciences educators to work together and stimulate the exchange of ideas and discussion and focus on the fourth degree of integration, *blending of basic science and clinical science*. The collaboration of educators in the development of course materials including learning objectives, patient cases, didactics, simulations and assessments resulted in instruction that included basic science that illustrates clinical relevance and clinical experiences that integrate basic science. This approach yielded deliberate integration of the basic and clinical sciences in the curriculum that may otherwise have been left to chance. For example, ISOM ran a simulation of a patient who presented to the ED unresponsive, the scenario goal was for the team to identify and treat an opioid overdose, and as part of the debrief, the team reviewed the neurobiology opiate overdose, the mechanism of action of narcan, and the physiology of breathing.

The considerations around the fifth degree of integration, *knowledge and skills-based progression*, led ISOM to create an objective structured clinical examination (OSCE) as a progress test. This OSCE included a station with a standardized patient who presents with a chief concern related to chronic pain. This progress test station was administered to students during each year/phase of their educational program. By engaging in the Arizona Curriculum on Pain and Addiction as described in the first four degrees of curricular integration, a progress test was able to demonstrate students’ advancement of knowledge and skills in identifying and treating pain and addiction as the integrated curriculum was presented to them over time.

The sixth degree of integration, *graduated responsibilities in the care of patients* with pain or addiction, is ISOM’s ultimate goal of its integrated curriculum on pain and addiction. The first five degrees of integration previously described prepare learners to put their knowledge and skills into direct delivery of care under appropriate supervision. At ISOM, required clinical experiences were incorporated into the emergency medicine clerkship that place a priority on health systems science. In that setting, an ISOM student would ideally have multiple opportunities, under direct observation of an attending or resident physician, to engage in patient care discussing risks and benefits of various pain management options, the impact of social determinants of health on pain management and opioid prescribing, providing counseling to patients with a lens on the structure of the healthcare system they are in, and receive feedback from their supervisor to develop an action plan for how to improve their patient care for the next patient.

In summary, by using the six degrees of curriculum integration as a model to define the phenomenon of integration, ISOM's effort results in a more comprehensive, multi-dimensional blending of the pain and addiction curriculum into their medical education program than would have been accomplished with a focus on horizontal and vertical integration alone.

References

1. The Arizona Pain and Addiction Curriculum [Internet]. Phoenix (AZ): Arizona Department of Health Services [cited 2024 Mar 30]. Available from: <https://www.azhealth.gov/curriculum>
